# Unveiling the Dynamics of NO_3_ at the Air–Water Interface and in Bulk Water: A Comparative Study with Cl and ClO

**DOI:** 10.3390/molecules30081724

**Published:** 2025-04-11

**Authors:** Yongxia Hu, Ying Zhou, Mohammad Hassan Hadizadeh, Fei Xu

**Affiliations:** Environment Research Institute, Shandong University, Qingdao 266237, China; 202232994@mail.sdu.edu.cn (Y.H.); 202333014@mail.sdu.edu.cn (Y.Z.)

**Keywords:** NO_3_, air–water interface, bulk water, AIMD, radicals

## Abstract

The interaction of nitrate radicals (NO_3_) with the air–water interface is a critical aspect of atmospheric chemistry, influencing processes such as secondary organic aerosol (SOA) formation, pollutant transformation, and nighttime oxidation. This study investigates the behavior of NO_3_ radicals at the air–water interface and in bulk water environments through ab initio molecular dynamics simulations, directly comparing them with Cl and ClO radicals. Three distinct configurations of NO_3_ in water droplets were analyzed: surface-parallel, surface-perpendicular, and bulk-phase. The results reveal environment-dependent dynamics, with surface-localized NO_3_ radicals exhibiting fewer but more flexible hydrogen bonds compared to bulk-solvated radicals. Analysis of radial distribution functions, coordination numbers, and population distributions demonstrates that NO_3_ radicals maintain distinct interfacial and bulk-phase preferences, with rapid equilibration in both environments. Electronic structure analysis shows significant modulation of spin density and molecular orbital distributions between surface and bulk environments. The comparative analysis with Cl and ClO radicals highlights how the unique planar geometry and delocalized π-system of NO_3_ influence its hydration patterns and interfacial activity. These results offer fundamental molecular-level insights into NO_3_ radical behavior at the air–water interface and in aqueous environments, enhancing our understanding of their role in heterogeneous atmospheric processes and nocturnal chemistry.

## 1. Introduction

Nitrate radical (NO_3_) is a significant nocturnal oxidant in the troposphere, primarily formed by the reaction of nitrogen dioxide (NO_2_) with ozone (O_3_) [1]. During the day, NO_3_ is rapidly photolyzed and reacts with NO, leading to very low daytime concentrations [1,2]. The presence of NO_3_ radicals is crucial for understanding the oxidative capacity of the atmosphere. They contribute to the removal of pollutants and the formation of secondary pollutants, such as ozone, through complex reaction pathway involving NOx [3]. At night, NO_3_ can react with NO_2_ to form dinitrogen pentoxide (N_2_O_5_), which is in thermal equilibrium with NO_3_ [2]. The lifetime of NO_3_ is influenced by relative humidity, temperature, and the presence of particulate matter [4,5]. High relative humidity and particulate matter concentrations enhance NO_3_ removal through indirect processes [4]. NO_3_ removal processes include photolysis, reaction with NO, and heterogeneous uptake on surfaces [1]. Meanwhile, the interactions between NO_3_ and various volatile organic compounds (VOCs) significantly affect its reactivity and removal rates. For instance, studies have shown that NO_3_ reacts with polycyclic aromatic hydrocarbons (PAHs) and their derivatives, leading to the formation of nitrated compounds that can have health implications [6]. Meanwhile, NO_3_ plays a crucial role in the nighttime oxidation of biogenic volatile organic compounds (BVOCs), leading to significant secondary organic aerosol (SOA) formation. For instance, studies show that NO_3_ reactions with monoterpenes like α-pinene and β-pinene produce highly oxygenated organic nitrates in both gas and particle phases [7]. The efficiency of nighttime biogenic SOA formation is comparable to daytime SOA formation from mixed anthropogenic and biogenic emissions, with mass yields up to 0.55 for isoprene oxidation by NO_3_ [8]. Organic nitrates can comprise 30–45% of the NO_y_ budget, with monoterpene nitrates contributing significantly to aerosol formation [9]. These findings highlight the importance of NO_3_-BVOC chemistry in nighttime SOA formation and atmospheric nitrogen cycling.

The kinetics of NO_3_ reactions with organic compounds have been extensively studied, revealing that while NO_3_ has a longer atmospheric lifetime compared to OH, its reactivity can still lead to the formation of toxic nitrated derivatives [10,11]. For example, the reaction of methoxyphenols with NO_3_ can produce oxalic acid, which is significant for aerosol formation and growth [12]. Additionally, the interaction of NO_3_ with particulate matter can lead to heterogeneous reactions that further complicate the atmospheric chemistry landscape [13]. The reactivity of NO_3_ is influenced by environmental conditions, such as temperature and humidity, which can affect the rates of these reactions and the overall atmospheric composition [14,15]. Furthermore, policy measures like China’s Clean Air Action Plan have been observed to influence NO_3_ levels and its nocturnal chemistry significantly. The implementation of this plan led to a reduction in primary pollutants and altered the rates of NO_3_ loss processes, thereby affecting the overall atmospheric chemistry related to NO_3_ [16]. Overall, the research on NO_3_ radicals highlights their importance as nocturnal oxidants in the troposphere, with implications for air quality and the chemical lifecycle of other atmospheric pollutants.

The air–water interface plays a crucial role in atmospheric chemistry as it serves as a dynamic boundary layer where the atmosphere interacts with aqueous phases such as oceans, lakes, cloud droplets, fog, and aerosols [17,18,19]. This interface is a unique microenvironment with distinct physical and chemical properties compared to bulk air or water, making it a hotspot for various chemical processes. At the air–water interface, molecules can exhibit altered reactivity due to differences in polarity, hydrogen bonding, and molecular orientation [20]. The interface facilitates the exchange of gases between the atmosphere and water, influencing processes like gas absorption, pollutant degradation, and the release of volatile species. It also provides a platform for heterogeneous and multiphase reactions, which are critical in the formation and aging of SOA, cloud condensation nuclei, and the cycling of reactive nitrogen and halogens [21]. Recent research on gas-liquid interfaces of nitrogen-containing substances like N_2_O_5_ interactions with aqueous surfaces showed that N_2_O_5_ readily adsorbs to water surfaces, with a 95% trapping probability and surface residence time of at least 20 ps [22]. Indeed, at the interface, N_2_O_5_ undergoes charge separation between NO_2_ and NO_3_ groups and experiences rapid energy accommodation [22]. Thus, studying radicals like NO_3_ at the air–water interface is particularly important because their behavior and reactivity at this interface differ significantly from those in bulk phases, directly affecting nighttime chemistry, SOA formation, and pollutant degradation.

Experimental studies on different species at the air–water interface have utilized various spectroscopic techniques such as second-harmonic generation (SHG) to investigate the adsorption of nitrate and other species at the fused quartz/water interface [23]. Electronic sum frequency generation spectroscopy has been utilized to examine the π → π* transition of nitrite at the air–water interface, challenging previous assumptions about interfacial adsorption [24]. However, such experimental approaches suffer from limitations, including difficulty in isolating interfacial contributions from bulk behaviors, low sensitivity to transient species, and challenges in resolving the structural orientation and dynamics of NO_3_ at the molecular level. These experimental shortages highlight the need for theoretical work, as computational models such as molecular dynamics simulations and quantum chemistry can complement experiments by providing molecular-level details of the NO_3_ radical’s orientation, hydrogen bonding interactions, and reaction mechanisms at the interface, which are inaccessible experimentally. Ab initio molecular dynamics (AIMD) simulations have proven to be a powerful tool for exploring radical behavior at the air–water interface. AIMD studies have revealed ion-specific effects and surface preferences of halide ions [25], as well as the tendency of Cl and ClO radicals to remain near the air–water interface in water droplet systems [18]. These simulations have also provided insights into the surface tension of water at the air–water interface, highlighting the importance of van der Waals corrections and basis set selection [26]. Furthermore, AIMD simulations are used to investigate chemical processes involving chlorine nitrate at the air–water interface, providing insights into reaction pathways and the role of halogen bonds [27]. These studies demonstrate the effectiveness of AIMD in elucidating molecular-scale structures and processes at interfaces, providing valuable benchmarks for improving simulations and understanding atmospheric and electrochemical phenomena.

This study systematically investigates the interactions between NO_3_ radicals and water molecules at the air–water interface using AIMD simulations. Indeed, this study provides an initial step toward understanding the dynamic behavior, hydration structures, and electronic properties of NO_3_ radicals at the air–water interface and in bulk aqueous environments. The dynamic behavior and mobility of NO_3_ radicals within water droplets were analyzed to provide a detailed understanding of their movement in an aqueous environment. To further characterize these interactions, radial distribution function (RDF) analysis was used to examine the formation of hydrogen bonds between NO_3_ radicals and water molecules. Coordination number and population analyses were also conducted to quantify the number of hydrogen bonds formed and to identify the most dominant configurations of NO_3_ radicals in the water droplet systems. Additionally, a comparative analysis was performed to evaluate the behavior of NO_3_ radicals at the air–water interface relative to other radicals, such as Cl and ClO. This comparison highlights the differences in mobility, bonding, and structural configurations between NO_3_ and Cl/ClO radicals in aqueous environments. The findings provide deeper insights into the unique physicochemical properties of NO_3_ radicals and their interactions at the air–water interface. This work aims to advance understanding of radical-driven oxidation processes in atmospheric chemistry.

## 2. Results and Discussion

**Figure 1:**
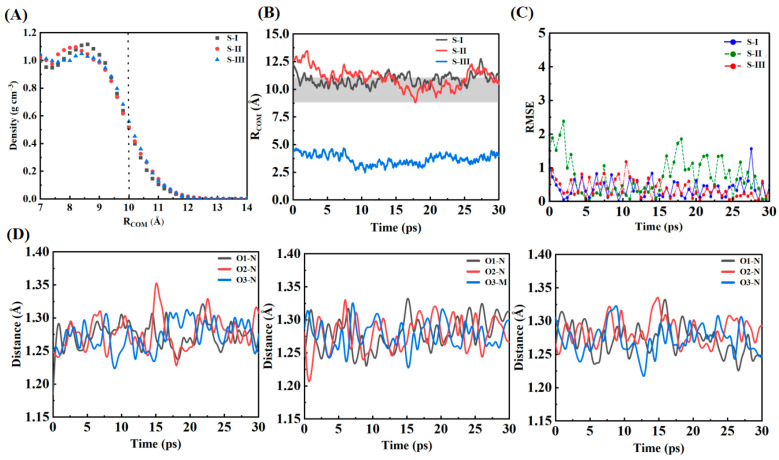
(**A**) Radial density profiles of water showing the air–water interface at ~10 Å (vertical dashed line) for S-I, S-II, and S-III. (**B**) Time evolution of NO_3_ distance from the COM, with interfacial preference (gray shaded area) in S-I/S-II and bulk localization in S-III. (**C**) RMSE fluctuations within 30 ps for all systems. (**D**) Bond length fluctuations (N–O1, N–O2, N–O3) for NO_3_ across S-I, S-II, and S-III, highlighting dynamic stability.

The trajectory analysis and equilibrium dynamics of NO_3_ radicals in water droplet systems reveal distinctive spatial and temporal patterns. Figure 1A illustrates the density profiles of water molecules as a function of radial distance from the center of mass (COM) for three distinct initial configurations (S-I, S-II, and S-III). The density profiles exhibit characteristic bulk-like behavior (~1.0 g/cm^3^) up to approximately 10 Å, followed by a steep decline through the interfacial region. The interface position, denoted by a vertical dotted line at ~10 Å, corresponds to the region where the density decreases to 50% of the bulk value. Notably, all three systems display nearly identical density distributions, indicating consistent droplet structure regardless of NO_3_ radical positioning. The time evolution of NO_3_-COM distances (R_COM_) over 30 ps simulation periods is presented in Figure 1B, where the gray shaded region demarcates the interfacial zone, providing a reference for evaluating the radical’s spatial distribution at equilibrium. Systems S-I and S-II, where NO_3_ radicals were initially positioned at the surface with parallel and perpendicular orientations, respectively, exhibited rapid equilibration within the first 5 ps of simulation. After this initial equilibration period, both systems maintained average distances of 10–12 Å from the COM, demonstrating strong interfacial preference.

The bond length analysis of the NO_3_ radical in configurations S-I (surface-parallel), S-II (surface-perpendicular), and S-III (bulk-phase) reveals distinct environment-dependent structural and dynamic behaviors. The mean bond lengths of N–O1, N–O2, and N–O3 in S-I are 1.272 Å, 1.282 Å, and 1.270 Å, respectively, indicating that the planar alignment of the radical at the air–water interface leads to slightly perturbed bond lengths due to reduced hydrogen bonding. In S-II, the mean bond lengths are 1.272 Å, 1.283 Å, and 1.271 Å, respectively, showing a slight increase compared to S-I because the perpendicular orientation facilitates stronger water interactions. In S-III, the mean bond lengths are 1.272 Å, 1.283 Å, and 1.270 Å, reflecting the most stabilized structure, as the bulk water environment provides a symmetric and dense hydration shell. The standard deviations of the bond lengths, which indicate bond flexibility, are highest in S-I, with values of 0.030 Å (N–O1), 0.030 Å (N–O2), and 0.029 Å (N–O3). This reflects the reduced hydrogen bonding at the interface, allowing for greater dynamic freedom. In S-II, the standard deviations are slightly smaller at 0.029 Å, 0.029 Å, and 0.028 Å for N–O1, N–O2, and N–O3, respectively, as the perpendicular orientation improves water interactions and reduces bond flexibility. In S-III, the standard deviations are the smallest, with values of 0.028 Å, 0.027 Å, and 0.027 Å, respectively, indicating that the strong hydration shell in the bulk phase restricts bond length fluctuations and provides enhanced stabilization. The peak-to-peak amplitudes, representing the range of bond length oscillations, are largest in S-I, with values of 0.134 Å (N–O1), 0.101 Å (N–O2), and 0.125 Å (N–O3). This is due to the weaker and fewer hydrogen bonds at the air–water interface, which allow for significant bond stretching and compression. In S-II, the peak-to-peak amplitudes are slightly reduced to 0.129 Å, 0.099 Å, and 0.12164 Å for N–O1, N–O2, and N–O3, respectively, as the perpendicular alignment enhances water interactions and limits oscillations. In S-III, the amplitudes are the smallest, with values of 0.126 Å, 0.097 Å, and 0.118 Å, respectively, as the dense and symmetric hydration shell in the bulk phase minimizes bond length oscillations and ensures structural stability. Overall, these results highlight the environment-dependent behavior of NO_3_ radicals. In S-I, the reduced hydration at the interface allows for greater bond flexibility and dynamic oscillations. In S-II, improved hydrogen bonding reduces bond variability, while in S-III, the dense and symmetric hydration environment provides the greatest stabilization, with minimal bond length fluctuations and oscillations. These findings emphasize the critical role of hydration environments in modulating the structural and dynamic properties of NO_3_ radicals, with implications for their chemical reactivity and stability in atmospheric processes.

Figure 1C illustrates that the S-I configuration (surface-parallel) exhibits relatively low fluctuations in RMSE because the parallel orientation of the NO_3_ radical minimizes its direct interaction with water molecules. By aligning parallel to the air–water interface, the radical reduces the number of accessible binding sites for hydrogen bonding. This limited interaction results in fewer and weaker hydrogen bonds, stabilizing its position at the surface and reducing variability in its hydration dynamics. The reduced hydration also decreases thermal motion, leading to more stable behavior compared to the other configurations. In contrast, the S-II configuration (surface-perpendicular) shows the highest fluctuations in RMSE. The perpendicular orientation of the NO_3_ radical increases its exposure to water molecules, allowing for more dynamic and transient hydrogen bonding interactions. Water molecules can interact with all three oxygen atoms of the radical, but the hydrogen bonds formed are weaker and more flexible due to the limited density and asymmetric hydration environment at the interface. This interplay of transient hydrogen bonding and thermal motion leads to pronounced fluctuations, reflecting the unstable and dynamic nature of this configuration. The S-II configuration represents an intermediate state, where the radical interacts more dynamically with water while still being constrained by the interfacial environment. The S-III configuration (bulk-phase) displays the lowest fluctuations in RMSE. In the bulk phase, the NO_3_ radical is fully embedded within a robust and symmetric three-dimensional hydration shell. This environment promotes stronger and more persistent hydrogen bonding with surrounding water molecules, which significantly stabilizes the radical. The enhanced hydration suppresses thermal motion and variability, resulting in the most stable configuration among the three. The strong hydrogen bonding network in the bulk phase also reduces the mobility of the radical, enhancing its structural stability. Overall, these observations highlight distinct hydration and dynamic behaviors of NO_3_ radicals in different configurations. S-I shows stabilization through minimized hydration, S-II exhibits pronounced fluctuations due to dynamic interfacial interactions, and S-III demonstrates the highest stabilization due to strong bulk-phase hydration. These findings emphasize the critical role of molecular orientation and hydration environment in modulating the chemical behavior and atmospheric reactivity of NO_3_ radicals.

Figure 1D illustrates the bond length fluctuations (in Å) between the nitrogen atom (N) and the three oxygen atoms (O1, O2, O3) of the NO_3_ radical over a 30 ps simulation period for the three configurations (S-I, S-II, and S-III). The panels from left to right correspond to S-I (surface-parallel), S-II (surface-perpendicular), and S-III (bulk-phase). In all configurations, the bond lengths exhibit small oscillations around their equilibrium values, reflecting the structural stability of the NO_3_ radical across different hydration environments. For the surface configurations (S-I and S-II), the fluctuations are slightly larger compared to the bulk configuration (S-III), indicating that the reduced hydrogen bonding at the air–water interface leads to greater freedom of motion. In S-I and S-II, the bond lengths show periodic deviations with occasional peaks, reflecting dynamic interactions with water molecules at the interface. In contrast, the bond length fluctuations in S-III are smaller and more consistent, demonstrating enhanced stabilization due to the stronger and more symmetric hydrogen bonding network in the bulk aqueous phase. The contour plots illustrating the bond angles (O1–N–O2, O2–N–O3, and O3–N–O1) and bond lengths (O1–N, O2–N, and O3–N) of NO_3_ radicals in different configurations are provided in Figure S1 of the Supplementary Materials, further highlighting the structural stability and hydration-dependent variations.

**Figure 2:**
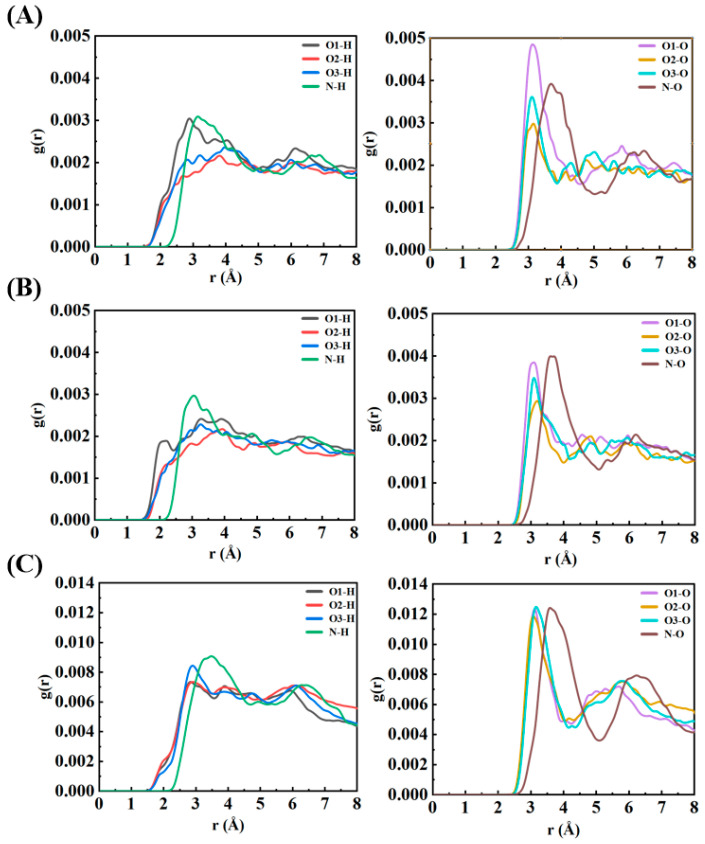
Radial distribution functions (RDFs) for NO_3_-water interactions in configurations S-I, S-II, and S-III. (**A**,**B**) show surface-bound hydration patterns with moderate hydrogen bond strengths and planar NO_3_ geometry. (**C**) depicts enhanced hydration structuring in the bulk phase with stronger and more symmetric hydrogen bonding networks.

The radial distribution function (RDF) analysis offers detailed insights into the spatial organization and bonding patterns of water molecules surrounding NO_3_ radicals in the three configurations studied: surface-parallel (S-I), surface-perpendicular (S-II), and bulk-phase (S-III). By examining the probability distribution of water molecules at various distances from the NO_3_ radical, the RDFs reveal how the hydration environment and molecular orientation influence the hydrogen bonding and solvation structure of NO_3_ (Figure 2). For the surface-bound configurations (S-I and S-II), the O-H correlations show prominent peaks in the range of 2–3 Å, representing direct hydrogen bonding between the oxygen atoms of NO_3_ (O_1_, O_2_, O_3_) and the hydrogen atoms of water molecules. These peaks indicate moderately strong hydrogen bonds, reflecting the planar geometry of NO_3_, which facilitates symmetrical interactions between its three oxygen atoms and surrounding water molecules. Additionally, the symmetry of the RDF peaks across all three oxygen atoms confirms that the planar π-system of NO_3_ is preserved at the air–water interface. The N-H correlations, on the other hand, are characterized by broader peaks at longer distances (above 3 Å), indicating weak and infrequent interactions between the nitrogen center of NO_3_ and water hydrogens. This weak interaction is attributed to the electron-withdrawing nature of the oxygen atoms, which reduces the electron density at the nitrogen site, as well as the steric hindrance created by the planar structure of NO_3_. These factors limit the nitrogen’s ability to act as a hydrogen bond acceptor. The O-O correlations display peaks at 3–4 Å, corresponding to the arrangement of water oxygen atoms in the vicinity of NO_3_. The presence of secondary peaks at 5–6 Å suggests the existence of a loosely ordered second hydration shell at the air–water interface. This less structured hydration environment at the interface arises from geometric constraints and reduced hydrogen bonding compared to the bulk phase.

In contrast, the bulk-phase configuration (S-III) exhibits sharper and more intense O-H peaks in the RDF, signifying stronger and more numerous hydrogen bonds between NO_3_ oxygen atoms and water molecules. The three-dimensional solvation environment in the bulk allows for a more symmetric and stabilized hydration shell around the NO_3_ radical. Furthermore, the O-O correlations in the bulk phase show pronounced second-shell peaks, indicative of a well-organized and cooperative hydrogen bonding network typical of bulk water. This extended hydration structure enhances the stabilization of NO_3_ in the bulk phase. Despite the significant differences in hydration strength and organization between the interface and bulk environments, the N-H correlations remain weak in the bulk phase, consistent with the behavior observed in the surface configurations. The nitrogen center of NO_3_ continues to contribute negligibly to hydrogen bonding interactions, regardless of the hydration environment.

Overall, the RDF analysis highlights the environment-dependent behavior of NO_3_ radicals. At the air–water interface (S-I and S-II), NO_3_ forms fewer and weaker hydrogen bonds, resulting in a more dynamic hydration structure. This flexibility promotes the radical’s interactions with gas-phase species, increasing its reactivity at the interface. In contrast, the bulk-phase configuration (S-III) demonstrates stronger and more extensive hydrogen bonding, which stabilizes the hydration shell and reduces the radical’s mobility and reactivity. These results underscore the critical role of hydration structures in modulating the chemical behavior of NO_3_ radicals. The planar geometry and delocalized π-system of NO_3_ facilitate symmetrical hydrogen bonding in both interfacial and bulk environments. However, the weaker hydration at the interface likely makes surface-localized NO_3_ radicals more accessible for interfacial chemical reactions, such as interactions with gas-phase pollutants. In comparison, the stronger and more structured hydration shell in the bulk phase electronically stabilizes NO_3_, reducing its reactivity and extending its atmospheric lifetime.

**Figure 3:**
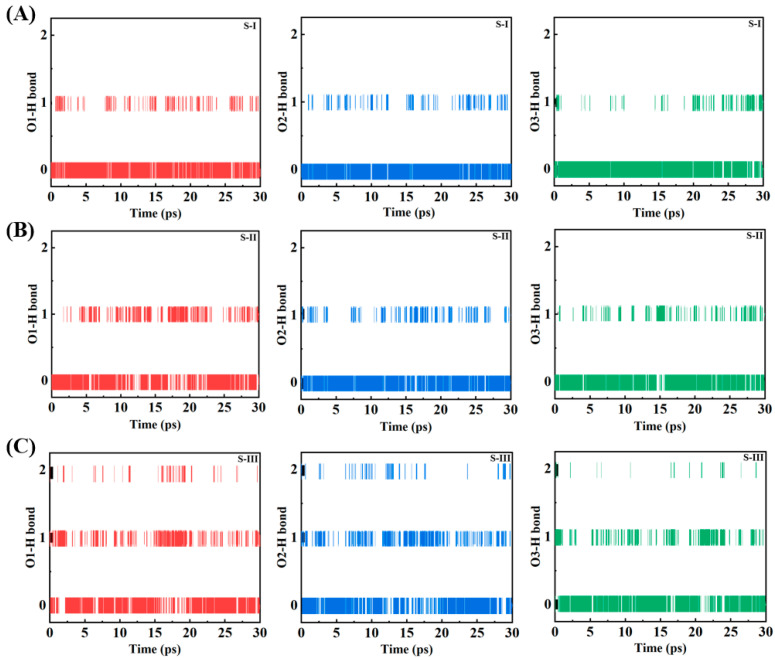
Temporal evolution of hydrogen bond coordination numbers for NO_3_ oxygen atoms (O1, O2, O3) during 30 ps simulations in configurations (**A**) S-I, (**B**) S-II, and (**C**) S-III. Surface configurations (S-I, S-II) show dynamic, fluctuating coordination states, while the bulk configuration (S-III) exhibits stable and sustained hydrogen bonding.

The temporal evolution of hydrogen bonding networks between NO_3_ radicals and water molecules is systematically analyzed through coordination number measurements across three distinct configurations (S-I, S-II, and S-III) during a 30 ps molecular dynamics simulation. The time series data presented in Figure 3 delineates the hydrogen bonding patterns for each oxygen atom (O1, O2, O3) of the NO_3_ radical, manifested through discrete coordination values of 0, 1, and 2, corresponding to the instantaneous number of hydrogen bonds. For surface-localized configurations (S-I and S-II, Figure 3A,B), the coordination states exhibit characteristic interfacial dynamics, predominantly oscillating between values of 0 and 1, with infrequent occurrences of double coordination events. The surface-bound species demonstrate pronounced temporal fluctuations in their hydrogen bonding networks, evidenced by rapid transitions between coordination states and extended periods of zero coordination, indicative of limited water accessibility at the interface. All three oxygen atoms manifest analogous hydrogen bonding patterns in the surface configurations, suggesting uniform exposure to the aqueous environment despite their spatial disposition. The bulk-phase configuration (S-III, Figure 3C) exhibits markedly distinct coordination dynamics, characterized by enhanced hydrogen bonding stability and increased frequency of double coordination events. The temporal evolution in the bulk phase reveals more sustained periods of single coordination, with notably fewer intervals of complete dehydration, reflecting the comprehensive solvation environment. The bulk configuration demonstrates more persistent hydrogen bonding patterns throughout the simulation period, with all three oxygen atoms exhibiting comparable behavior and enhanced stability in their hydration networks. There was no evidence of hemibond formation across all systems, as demonstrated in Figure S2. These coordination patterns provide molecular-level evidence for the environment-dependent hydration behavior of NO_3_ radicals, with significant implications for their reactivity and transport in atmospheric water droplets. The enhanced stability of hydrogen bonding networks in bulk-phase NO_3_ radicals, compared to their surface-bound counterparts, suggests fundamental differences in chemical reactivity and atmospheric residence times. The observed coordination dynamics contribute essential insights into the molecular mechanisms underlying heterogeneous atmospheric processes, particularly relevant to aerosol aging and atmospheric chemical transformations. These molecular-level observations establish a quantitative foundation for understanding NO_3_ radical behavior in atmospheric water droplets and enhance our comprehension of heterogeneous atmospheric chemistry.

**Figure 4:**
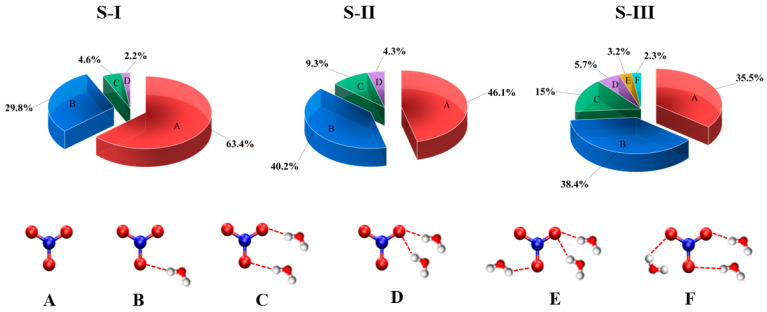
Population distributions of NO_3_(H_2_O)_n_ structures in configurations S-I, S-II, and S-III. Surface configurations (S-I, S-II) exhibit dominant non-hydrated and single-water coordination states, while the bulk configuration (S-III) features reduced non-hydrated populations and increased complex hydration patterns. A is non-hydrated and B to F are related to hydrated configurations.

The population distribution of NO_3_(H_2_O)_n_ structures across configurations S-I, S-II, and S-III reveals distinct hydration behaviors influenced by the radical’s orientation and environment, as illustrated in the Figure 4. In S-I (surface-parallel), the NO_3_ radical exhibits a dominant non-hydrated population (A: 63.4%) due to its planar geometry and amphiphilic nature, which minimizes direct hydration. Single-water coordination (B: 29.8%) is the most prominent hydration pattern, reflecting the formation of hydrogen bonds between the electron-rich oxygen atoms of NO_3_ and surface water hydrogens. Complex hydration patterns (C: 4.6%) occur less frequently, as steric hindrance and limited water accessibility at the surface restrict the formation of multiple simultaneous hydrogen bonds. In S-II (surface-perpendicular), the hydration behavior transitions toward greater water accessibility compared to S-I. The non-hydrated population (A: 46.1%) decreases, while single-water coordination (B: 40.2%) increases as the perpendicular orientation of the radical allows for better exposure to water molecules. Complex hydration patterns (C: 9.3%; D: 4.3%) are more prominent than in S-I as the perpendicular alignment facilitates improved interaction with both surface and bulk water molecules. However, the hydration in S-II remains constrained by the interfacial environment, preventing the formation of more extensive hydration patterns. In S-III (bulk-phase), the hydration structure becomes fully developed due to the three-dimensional accessibility of bulk water molecules. The non-hydrated population (A: 35.5%) is significantly reduced, while single-water coordination (B: 38.4%) remains prominent. Complex hydration patterns (C: 15%; D: 5.7%) are much more prevalent than in the surface configurations, as the bulk water environment allows for extended hydrogen bonding networks. The absence of interfacial constraints in S-III enables the radical to form a more stable and symmetric hydration structure, in consistent with contour plots of the angular orientation (θ), as shown in Figure S3.

**Figure 5:**
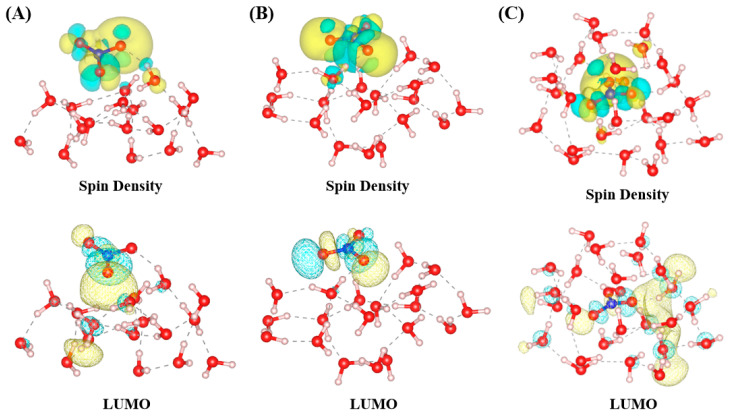
Spin density and LUMO (lowest unoccupied molecular orbital) distributions of NO_3_ radicals across hydration environments (surface and bulk) for (**A**) surface-parallel, (**B**) surface-perpendicular, and (**C**) bulk-phase. Surface configurations exhibit localized spin density and limited delocalization, while bulk environments facilitate increased electronic delocalization through extended hydrogen bonding networks.

**Figure 6:**
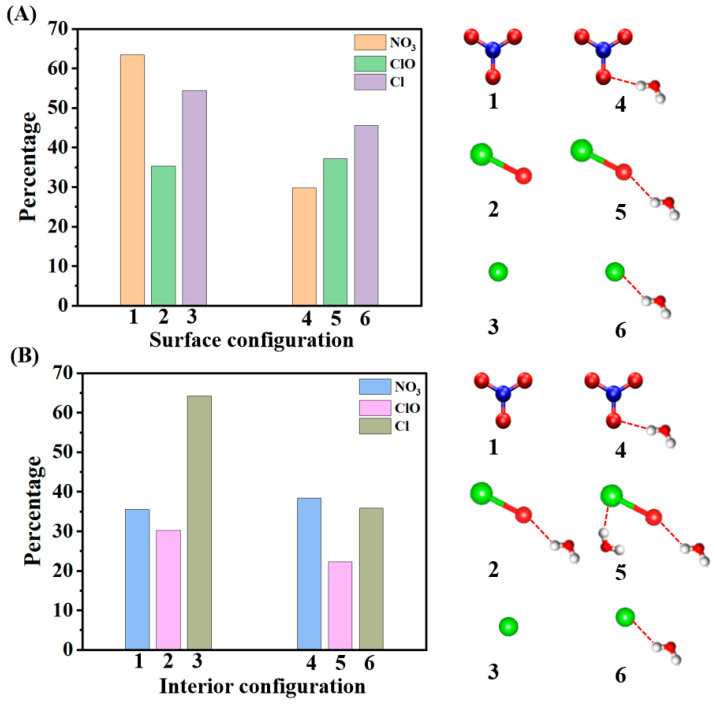
Population analysis of NO_3_, ClO, and Cl radical hydration states. (**A**) Surface and (**B**) interior distribution percentages of hydration configurations. Molecular diagrams (1–6) show representative structures: first high probability (1–3) and second high probability (4–6). Red dashed lines indicate hydrogen bonds. Color scheme: nitrogen (blue), oxygen (red), chlorine (green), and hydrogen (white). The data for Cl and ClO is derived from our previous work [18].

The spatial distribution of the highest occupied molecular orbital (HOMO) and lowest unoccupied molecular orbital (LUMO) plays a crucial role in determining the chemical reactivity of the NO_3_ radical. The localized nature of the LUMO enhances site-specific interactions with electron-donating species, particularly in interfacial environments where hydration is less structured and dynamic. This alignment between orbital localization and chemical reactivity is consistent with the principles of frontier molecular orbital theory, which highlights the importance of HOMO-LUMO interactions in governing chemical reactivity [28]. The provided figure (Figure 5) displays the spin density and LUMO distributions of NO_3_ radicals across different hydration environments: surface-parallel (A), surface-perpendicular (B), and bulk-phase (C). The isosurface plots for spin density and the LUMO in Figure 5 were generated using contour values of 0.0006 and 0.03 e/Å^3^, respectively, to ensure clear visualization of their spatial distributions. These plots were derived from snapshots of the most stable configuration after 10 ps of molecular dynamics equilibration, ensuring that the analyzed geometries were fully relaxed and representative of the system’s equilibrium state. These visualizations highlight key differences in electronic structure and hydration effects, which are closely tied to the conclusions about the NO_3_ radical’s behavior and reactivity in atmospheric systems. In the surface-parallel configuration (A), the spin density is primarily localized around the NO_3_ radical’s π* orbital, with minimal delocalization due to the limited hydrogen bonding and low water density at the air–water interface. The LUMO is similarly constrained, showing weak interaction with the surrounding water molecules. This electronic localization reflects the reduced hydration environment at the surface, which enhances the radical’s reactivity and accessibility for gas-phase or interfacial chemical reactions. In the surface-perpendicular configuration (B), the spin density begins to show moderate delocalization as the NO_3_ radical interacts more effectively with water molecules through hydrogen bonding. The LUMO distribution demonstrates increased overlap with the hydration shell, indicating stronger electronic coupling compared to configuration (A). This intermediate hydration environment allows for a balance between interfacial reactivity and stabilization. In the bulk-phase configuration (C), the spin density is extensively delocalized across the NO_3_ radical and its surrounding hydrogen bonding network. The LUMO distribution is highly integrated with the hydration shell, reflecting the dense and symmetric hydrogen bonding environment. This enhanced hydration stabilizes the radical and reduces its reactivity, consistent with the conclusions that bulk-solvated NO_3_ radicals are less reactive and exhibit extended atmospheric lifetimes compared to their surface-localized counterparts. These observations from Figure 5 align with this study’s conclusions by demonstrating how the hydration environment directly influences the electronic structure and reactivity of NO_3_ radicals. Surface-localized radicals (A and B) exhibit limited hydration and localized electronic states, making them more reactive at the interface. In contrast, bulk-phase radicals (C) are stabilized by stronger hydrogen bonding and delocalized electronic structures, reducing their reactivity and enhancing their atmospheric persistence. This relationship between hydration, electronic structure, and reactivity underscores the critical importance of interfacial and bulk-phase environments in determining the chemical behavior of NO_3_ radicals in atmospheric processes. For comparison, the HOMO orbitals are illustrated in Figure S4. Note that the HOMO of the NO_3_ radical in Figure S4 is not of π symmetry as it does not exhibit a nodal plane on the nuclei. Instead, it is a non-bonding orbital primarily localized on the oxygen atoms. This is consistent with the electronic structure of the NO_3_ radical, where the highest occupied molecular orbital is dominated by lone-pair contributions from the oxygen atoms. The spatial distributions of the HOMO and LUMO in Figure S5 and Figure 6 do not show significant differences in localization, suggesting that the apparent localization of the LUMO may be a byproduct of the Kohn–Sham calculation and visualization threshold. It is also important to note that the energies of the virtual orbitals, including the LUMO, are not physically accurate, as discussed in the Computational Details.

Figure 6 illustrates the comparative hydration behavior of NO_3_, ClO, and Cl radicals in surface and interior environments of water droplets, revealing distinctive population distributions governed by their electronic and structural properties. At the surface (Figure 6A), NO_3_ demonstrates a dominant non-hydrated configuration (~63%, Configuration 1) due to its planar geometry and delocalized π-system, where the negative charge density preferentially orients toward the bulk water while minimizing surface hydration to reduce surface tension. In contrast, the ClO radical shows a moderate single-water interaction (~35%, Configuration 2), which arises from its strong dipole moment and the electronegative oxygen atom’s ability to form directed hydrogen bonds with surface water molecules. Unlike NO_3_ and ClO, Cl exhibits the highest population in a weakly hydrated state (~53%, Configuration 3), driven by its spherical electron distribution and weaker hydration requirements. This distinction highlights that NO_3_ prefers minimal hydration at the interface due to its planar structure, ClO strikes a balance between hydration and interfacial activity, and Cl remains the least hydrated due to its isotropic electronic properties. In the droplet interior (Figure 6B), the hydration patterns of all three radicals shift due to the three-dimensional water accessibility. NO_3’_s reduced non-hydrated population (~35%) reflects its complete solvation accessibility, with symmetric and complex hydration structures forming due to its multiple interaction sites. ClO, while maintaining a similar behavior to its surface configuration (~30% non-hydrated), exhibits enhanced multiple water coordination, facilitated by its linear geometry and strong dipole interactions. Cl, on the other hand, demonstrates the highest interior population (~63%) through purely electrostatic interactions, forming uniform hydration shells that are less complex than those of NO_3_. Comparing the three radicals, NO_3_ exhibits the most dramatic transition from surface to interior, transitioning from minimal surface hydration to complex and symmetric hydration in the bulk. ClO shows moderate changes in its hydration behavior, maintaining directional hydrogen bonding across environments, while Cl exhibits the least variation, retaining its uniform spherical hydration patterns in both surface and bulk phases. These differences in hydration patterns are governed by several key factors: electronic structure, hydrogen bonding capabilities, geometric constraints, and surface tension effects.

NO_3_ forms extended hydrogen bonding networks in the bulk due to its delocalized negative charge and planar trigonal geometry, while at the surface, its geometry minimizes hydration for reduced surface tension. ClO exhibits directional hydrogen bonding from its linear geometry and dipole moment, which allows it to adapt moderately between surface and bulk environments. Cl, with its spherical charge distribution, exhibits consistent and isotropic hydration patterns, highlighting its weaker interactions with water. Overall, NO_3_ demonstrates the most dramatic surface-to-bulk transition, with non-hydrated configurations decreasing from 63.4% to 35.5%, highlighting its environment-dependent behavior. These distinct hydration patterns significantly influence the radicals’ atmospheric reactivity and transport.

## 3. Computational Details

**Figure 7:**
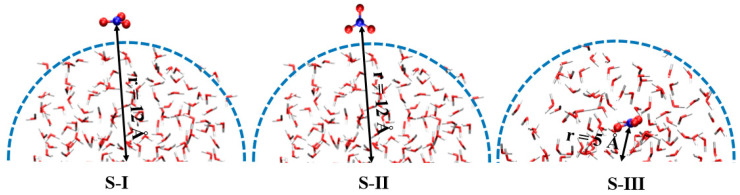
Initial configurations (S-I, S-II, S-III) of NO_3_ radicals in water droplet systems. S-I: NO_3_ radical positioned parallel to the droplet surface; S-II: NO_3_ radical aligned perpendicularly to the surface; S-III: NO_3_ radical embedded in the droplet interior.

The behavior of NO_3_ radicals at the air–water interface was investigated using three distinct configurations of spherical water droplets, each with an initial radius of 10 Å, containing 191 H_2_O molecules and 1 NO_3_ radical. While the initial droplet radius was set to 10 Å, the system naturally expanded to approximately 12 Å after equilibration through DFT and AIMD simulations (~3 ps), resulting in a realistic density distribution that captures both bulk-like and interfacial behaviors. These configurations, labeled S-I, S-II, and S-III, represent different spatial and orientational arrangements of the radical, as shown in Figure 7. In S-I, the NO_3_ radical is positioned on the droplet surface, oriented parallel to the interface, with its nitrogen atom closest to the water surface and approximately 12 Å from the droplet’s center. In S-II, the radical is also located on the surface but aligned perpendicularly to the interface, maintaining the same center-to-nitrogen distance of 12 Å. In S-III, the NO_3_ radical is embedded inside the droplet, approximately 5 Å from the center, representing a bulk water environment. These setups were chosen to systematically examine the structural, electronic, and dynamic properties of NO_3_ radicals in both interfacial and bulk water regions. To ensure the physical accuracy of the simulations and avoid artificial interactions between periodic images, each droplet was placed in a cubic simulation box with dimensions of 40 Å. The NO_3_ radical was first optimized using density functional theory (DFT) to obtain an accurate structure before being embedded into the droplets. A Gaussian basis set was employed to represent the wavefunctions, complemented by an auxiliary plane wave basis set to improve computational efficiency [29,30]. Ab initio molecular dynamics (AIMD) simulations were then performed using the Born–Oppenheimer molecular dynamics (BOMD) approach implemented in the Quickstep module of the CP2K package version 2024.3 [31], which has been effectively utilized in previous studies [32,33,34]. The DFT calculations used the hybrid Becke (B) exchange functional combined with the Lee–Yang–Parr (LYP) correlation functional (B3LYP) to describe the electronic structure of the systems [35]. Goedecker–Teter–Hutter (GTH) pseudopotentials were applied [31], with a double-zeta valence polarized (DZVP) basis set for high accuracy. Weak dispersion interactions, critical for modeling NO_3_-water interactions, were accounted for using Grimme’s DFT-D3 dispersion correction with zero damping [36]. Following geometry optimization, all systems were equilibrated for 400 fs using BOMD simulations to ensure stability (Figure S5). Production runs were conducted under a canonical (NVT) ensemble, with the system temperature (300 K) controlled by the Nose–Hoover chain thermostat with a time constant of 0.1 ps. The timestep for integration was set to 0.5 fs to ensure stability and precision over the course of the simulations. It should be noted that within AIMD simulations, no implicit solvent model was employed. Instead, solvent effects were explicitly included by embedding the NO_3_ radical in spherical water droplets containing 191 water molecules. This explicit solvation model allowed us to capture the dynamic interactions and hydrogen bonding between the NO_3_ radical and surrounding water molecules in three configurations: surface-parallel, surface-perpendicular, and bulk-phase. The explicit treatment of the solvent provided a detailed and realistic description of the hydration environment, enabling us to investigate interfacial and bulk-phase effects at the molecular level [37,38]. While implicit solvent models can approximate bulk solvation effects, they are less suitable for capturing the detailed interfacial dynamics and specific hydrogen bonding interactions, which are central to this study. Therefore, explicit solvation was used to ensure accurate modeling of the hydration-dependent structural and electronic properties of NO_3_ radicals. Meanwhile, the coordination number measurements were calculated based on the number of water molecules forming hydrogen bonds with each oxygen atom of the NO_3_ radical. A hydrogen bond was defined using a geometric criterion: the distance between the oxygen atom of the NO_3_ radical and the hydrogen atom of a water molecule was less than 2.1 Å, and the angle between the O(NO_3_)–H–O(H_2_O) atoms was less than 30°. These criteria were chosen based on standard hydrogen bonding definitions in molecular dynamics simulations of aqueous systems. Spin-polarized calculations were included to account for the unpaired electron on the NO_3_ radical during its interactions with water molecules. This computational strategy provides a robust methodology for analyzing NO_3_ radicals in different configurations relative to the water droplet. The systematic exploration of the radical’s properties in bulk and interfacial environments yields valuable insights into its structural, electronic, and dynamic behavior at the air–water interface. It is important to note that the virtual orbital energies, such as the LUMO, computed using Kohn–Sham DFT, do not correspond directly to the true electron affinities of the system. These orbital energies are often underestimated due to the approximate nature of exchange–correlation functionals, such as B3LYP, and their inability to fully account for electron correlation and self-interaction errors. Thus, it is advised to interpret these energies qualitatively rather than quantitatively.

## 4. Conclusions

This study provides a comprehensive investigation into the dynamic behavior, hydration structures, and electronic properties of NO_3_ radicals at the air–water interface and in bulk aqueous environments using ab initio molecular dynamics (AIMD) simulations. By systematically analyzing the spatial distribution, radial distribution functions (RDFs), hydrogen bonding networks, and electronic structures of NO_3_ radicals in three distinct configurations (surface-perpendicular, surface-parallel, and bulk-phase), we have uncovered several critical insights into their physicochemical properties and atmospheric implications.

The results reveal that NO_3_ radicals exhibit distinct interfacial and bulk-phase preferences, with rapid equilibration and stable configurations observed in both environments. Surface-localized NO_3_ radicals maintain fewer but more flexible hydrogen bonds, which could facilitate potential interactions with gas-phase species and contribute to further heterogeneous reactions. In contrast, bulk-solvated radicals experience enhanced hydration and stronger hydrogen bonding networks, which stabilize their electronic structure and reduce their reactivity. The electronic analysis highlights a significant environment-dependent modulation of the radical’s spin density and molecular orbital distributions, with bulk-phase hydration promoting greater delocalization of the radical’s π-system compared to surface-localized configurations.

The comparative analysis reveals distinct patterns in radical-surface interactions. NO_3_ demonstrates the strongest surface preference with 63.4% non-hydrated surface configurations, followed by Cl (53%) and ClO (35%). In terms of hydrogen bonding, NO_3_ forms the most complex and extensive networks in bulk water (up to 3–4 hydrogen bonds) but shows limited hydration at the surface. ClO maintains moderate hydrogen bonding (2–3 bonds) across both environments, while Cl exhibits the weakest but most uniform hydration patterns. NO_3_ undergoes the most significant transition between surface and bulk environments, showing a dramatic decrease in non-hydrated configurations from 63.4% to 35.5%, indicating its highly environment-dependent behavior. These distinct patterns in surface affinity and hydrogen bonding networks directly influence their atmospheric reactivity and transport properties.

Overall, this work provides a molecular-level understanding of NO_3_ radical behavior in aqueous environments, offering valuable insights into their role in atmospheric processes, including atmospheric nitrogen cycles, secondary organic aerosol formation, pollutant transformation, and nocturnal oxidation chemistry. The findings not only highlight the importance of interfacial chemistry in modulating radical reactivity but also provide critical benchmarks for improving atmospheric models and designing future experimental studies aimed at elucidating the behavior of reactive species in heterogeneous environments. While this work focuses on the unreacted NO_3_ radical, we acknowledge that chemical reactions involving NO_3_ could influence its behavior and properties. Building on the foundational insights provided here, future work will explore the explicit chemical reactivity of NO_3_ radicals with relevant atmospheric species to further elucidate their role in heterogeneous atmospheric processes such as secondary organic aerosol (SOA) formation and pollutant transformation.

## Data Availability

Data will be made available on request.

## Supplementary Materials

The following supporting information can be downloaded at https://www.mdpi.com/article/10.3390/molecules30081724/s1: Figure S1: Time evolution of bond distances (O1–N, O2–N, and O3–N) during BOMD simulations for systems S-I, S-II, and S-III. Each system was equilibrated for 400 fs following geometry optimization, demonstrating stable oscillatory behavior and structural stability; Figure S1: Contour plots of the bond angles (O1–N–O2, O2–N–O3, and O3–N–O1) and bond lengths (O1–N, O2–N, and O3–N) of the NO_3_ radical in three distinct environments: (A) surface-parallel (S-I), (B) surface-perpendicular (S-II), and (C) bulk-phase (S-III). The plots highlight the structural stability of the NO_3_ radical, with slight differences in bond angles and lengths influenced by the hydration environment. The bulk configuration (C) exhibits tighter distributions, indicating enhanced stabilization compared to interfacial configurations (A and B); Figure S2: Time evolution of O(NO)–O(H_2_O) distances (O1–O, O2–O, and O3–O) between the oxygen atoms of the NO_3_ radical and surrounding water molecules in three configurations: (A) surface-parallel (S-I), (B) surface-perpendicular (S-II), and (C) bulk-phase (S-III). The results confirm the absence of hemibond formation across all configurations; Figure S3: Contour plots of the angular orientation (θ) of the NO_3_ radical relative to the center of mass (COM) of the water droplet as a function of distance (d_n_,COM) in three configurations: (A) surface-parallel (S-I), (B) surface-perpendicular (S-II), and (C) bulk-phase (S-III). The illustrations below each plot depict the orientation of the NO_3_ radical with respect to the droplet interface or interior. The surface configurations (S-I and S-II) show distinct angular preferences, while the bulk-phase configuration (S-III) exhibits a more stabilized orientation due to symmetric hydration; Figure S4: Visualization of the Highest Occupied Molecular Orbital (HOMO) for three different molecular configurations S-I, S-II, and S-III (from left to right) interacting with surrounding water molecules. The HOMO is represented by the colored orbital lobes, while the hydrogen bonding network of water molecules is shown with dashed lines; Figure S5: Time evolution of bond distances (O1–N, O2–N, and O3–N) during BOMD simulations for systems S-I, S-II, and S-III. Each system was equilibrated for 400 fs following geometry optimi-zation, demonstrating stable oscillatory behavior and structural stability
