# Supplementary material for: Unveiling the Dynamics of NO3 at the Air–Water Interface and in Bulk Water: A Comparative Study with Cl and ClO
Source: Molecules. 2025 Apr 11;30(8):1724. doi: 10.3390/molecules30081724 (PMC12029586; doi:10.3390/molecules30081724)
Supplement: Supplementary file 1 [file molecules-30-01724-s001.zip › molecules-3526039-supplementary - updated.pdf]

Supplementary Information for

Unveiling the Dynamics of NO3 at the Air–Water Interface and in Bulk Water: A Comparative Study with Cl and ClO

Yongxia Hu, Ying Zhou, Mohammad Hassan Hadizadeh * and Fei Xu *

Environment Research Institute, Shandong University, Qingdao 266237, China;

202232994@mail.sdu.edu.cn (Y.H.); 202333014@mail.sdu.edu.cn (Y.Z.)

*Correspondence: hadizadeh.mh@sdu.edu.cn (M.H.H.); xufei@sdu.edu.cn (F.X.);

Tel.: +86-532-58631992 (M.H.H. & F.X.)

Contains five figures

Figure S1. Contour plots of the bond angles (O1-N-O2, O2-N-O3, and O3-N-O1) and bond lengths (O1-N, O2-N, and O3-N) of the NO_3 radical in three distinct environments: (A) surface-parallel (S-I), (B) surface-perpendicular (S-II), and (C) bulk-phase (S-III). The plots highlight the structural stability of the NO_3 radical, with slight differences in bond angles and lengths influenced by the hydration environment. The bulk configuration (C) exhibits tighter distributions, indicating enhanced stabilization compared to interfacial configurations (A and B).

Figure S2. Time evolution of O(NO)–O(H_2O) distances (O1–O, O2–O, and O3–O) between the oxygen atoms of the NO_3 radical and surrounding water molecules in three configurations: (A) surface-parallel (S-I), (B) surface-perpendicular (S-II), and (C) bulk-phase (S-III). The results confirm the absence of hemibond formation across all configurations.

Figure S3. Contour plots of the angular orientation (θ) of the NO_3 radical relative to the center of mass (COM) of the water droplet as a function of distance (d_n,COM) in three configurations: (A) surface-parallel (S-I), (B) surface-perpendicular (S-II), and (C) bulk-phase (S-III). The illustrations below each plot depict the orientation of the NO_3 radical with respect to the droplet interface or interior. The surface configurations (S-I and S-II) show distinct angular preferences, while the bulk-phase configuration (S-III) exhibits a more stabilized orientation due to symmetric hydration.

Figure S4. Visualization of the Highest Occupied Molecular Orbital (HOMO) for three different molecular configurations S-I, S-II, and S-III (from left to right) interacting with surrounding water molecules. The HOMO is represented by the colored orbital lobes, while the hydrogen bonding network of water molecules is shown with dashed lines.

Figure S5. Time evolution of bond distances (O1–N, O2–N, and O3–N) during BOMD simulations for systems S-I, S-II, and S-III. Each system was equilibrated for 400 fs following geometry optimization, demonstrating stable oscillatory behavior and structural stability.

.

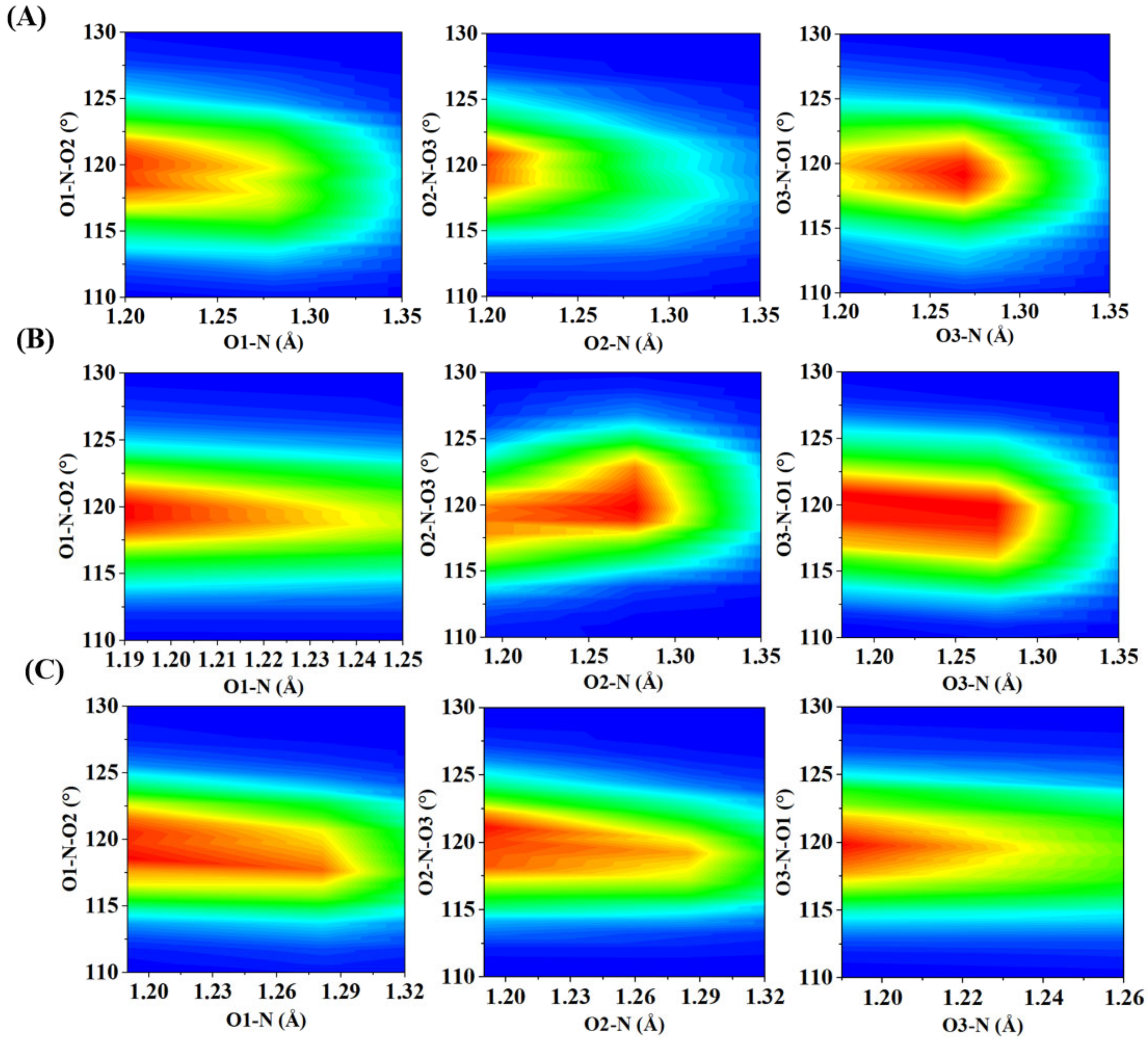

Figure S1. Contour plots of the bond angles (O1-N-O2, O2-N-O3, and O3-N-O1) and bond lengths (O1-N, O2-N, and O3-N) of the NO_3 radical in three distinct environments: (A) surface-parallel (S-I), (B) surface-perpendicular (S-II), and (C) bulk-phase (S-III). The plots highlight the structural stability of the NO_3 radical, with slight differences in bond angles and lengths influenced by the hydration environment. The bulk configuration (C) exhibits tighter distributions, indicating enhanced stabilization compared to interfacial configurations (A and B).

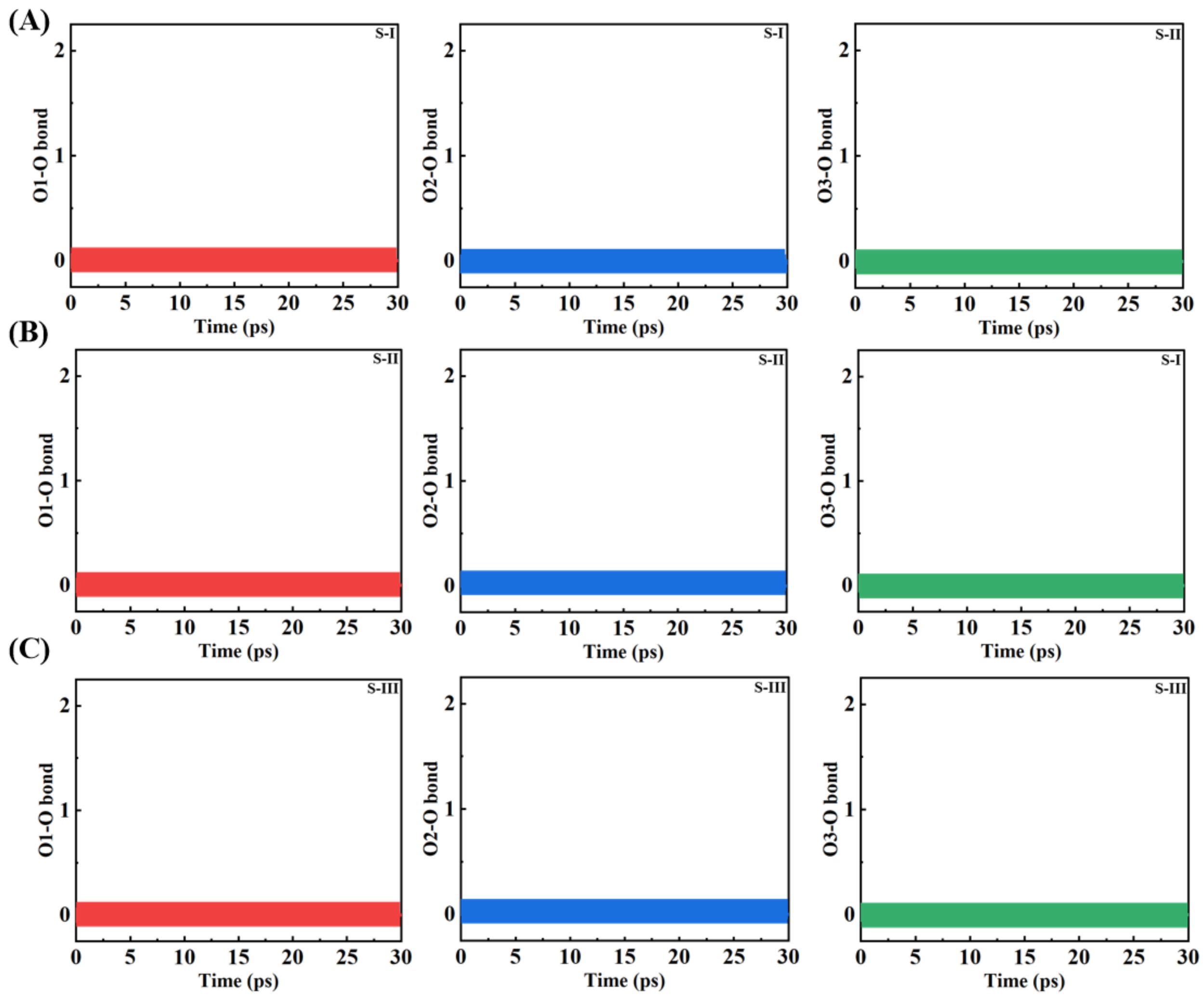

Figure S2. Time evolution of O(NO)–O(H_2O) distances (O1–O, O2–O, and O3–O) between the oxygen atoms of the NO_3 radical and surrounding water molecules in three configurations: (A) surface-parallel (S-I), (B) surface-perpendicular (S-II), and (C) bulk-phase (S-III). The results confirm the absence of hemibond formation across all configurations.

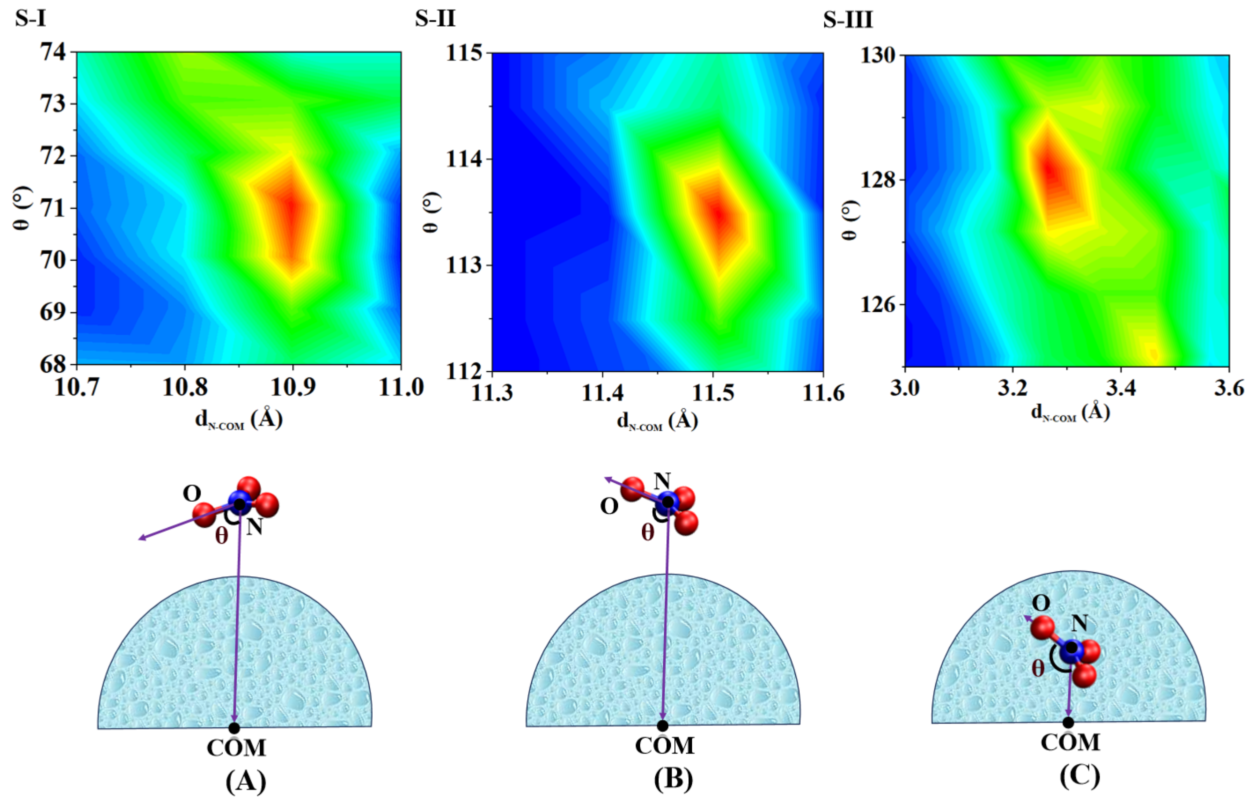

Figure S3. Contour plots of the angular orientation (θ) of the NO_3 radical relative to the center of mass (COM) of the water droplet as a function of distance (d_n,COM) in three configurations: (A) surface-parallel (S-I), (B) surface-perpendicular (S-II), and (C) bulk-phase (S-III). The illustrations below each plot depict the orientation of the NO_3 radical with respect to the droplet interface or interior. The surface configurations (S-I and S-II) show distinct angular preferences, while the bulk-phase configuration (S-III) exhibits a more stabilized orientation due to symmetric hydration.

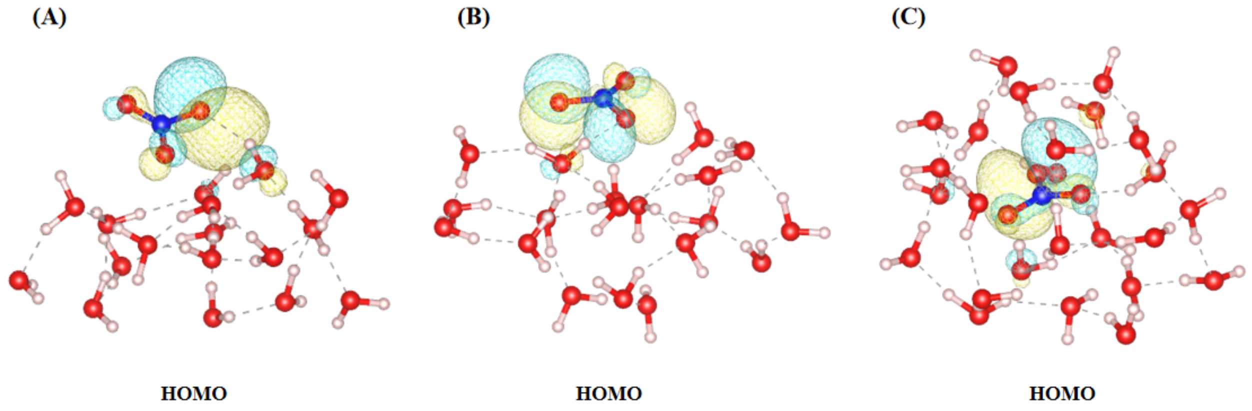

Figure S4. Visualization of the Highest Occupied Molecular Orbital (HOMO) for three different molecular configurations S-I, S-II, and S-III (from left to right) interacting with surrounding water molecules. The HOMO is represented by the colored orbital lobes, while the hydrogen bonding network of water molecules is shown with dashed lines.

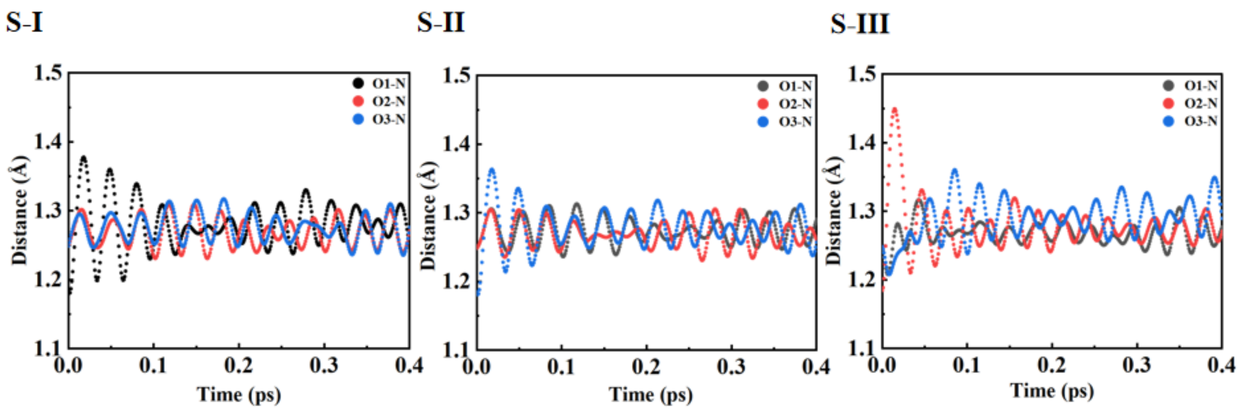

Figure S5. Time evolution of bond distances (O1–N, O2–N, and O3–N) during BOMD simulations for systems S-I, S-II, and S-III. Each system was equilibrated for 400 fs following geometry optimization, demonstrating stable oscillatory behavior and structural stability
